# Four complete genomes of human parvovirus B19 from amniotic fluid specimens

**DOI:** 10.1128/MRA.00556-23

**Published:** 2023-09-15

**Authors:** Enagnon Kazali Alidjinou, Lander De Coninck, Jill Swinnen, Mouna Lazrek, Didier Hober, Jelle Matthijnssens

**Affiliations:** 1 Univ Lille, CHU de Lille, Laboratoire de Virologie ULR 3610, Lille, France; 2 KU Leuven, Department of Microbiology, Immunology and Transplantation, Rega Institute, Laboratory of Viral Metagenomics, Leuven, Belgium; DOE Joint Genome Institute, Berkeley, California, USA

**Keywords:** parvovirus B19, genomes, amniotic fluid

## Abstract

We report the sequences of four complete genomes of parvovirus B19, extracted from human amniotic fluid specimens collected from pregnant women with abnormal ultrasound features in France. The genome sequences are 5,596 nucleotides long and include long terminal repeats. Several amino acid substitutions were observed in nonstructural protein (NS1).

## ANNOUNCEMENT

Parvovirus B19 belongs to the species *Erythroparvovirus primate 1*, in the genus *Erythroparvovirus* in the family *Parvoviridae* (1). Clinically, B19 is associated with a wide range of diseases, such as “erythema infectiosum” in children, acute polyarthritis in adults, and aplastic crisis in sickle cell disease patients. Primary infection in pregnant women can cause hydrops fetalis in the developing second-trimester fetus. The diagnosis of congenital infection is usually confirmed by testing amniotic fluid samples (AFS) (2). The B19 genome is a single-stranded DNA (ssDNA) molecule of 5,596 nucleotides (nt) with long (383 nt) terminal repeats (TRs). Three genotypes (G) are currently described, including the widely circulating G1 and two others—G2 and G3—which diverge in genome nucleotide sequence by ~10% (1, 3). Sequences of several isolates from human samples are available. However, no sequence detected from AFS has been reported.

Five AFS (P1–P5) collected between 2017 and 2019 at the University Hospital of Lille, France, through amniocentesis from pregnant women presenting with abnormal fetal ultrasound features tested positive for B19 by a qPCR assay (AltoStar Parvovirus B19 PCR Kit, Altona Diagnostics). The viral loads were 6.4, 8.1, 2.8, 8.6, and 8.1 Log copies/mL for P1–P5, respectively. Samples were stored at −80°C and later used for sequencing.

We performed the previously described NetoVIR protocol for viral particle purification (4, 5). Briefly, 200 µL of AFS were enriched for virus-like particles (centrifugation, filtration, and nuclease treatment) and submitted to nucleic acid extraction using the QIAamp Viral RNA Mini Kit (Qiagen). Then, cDNA synthesis and random PCR amplification were done using the whole transcriptome amplification kit 2 (Sigma Aldrich). Finally, library preparation using the Nextera XT DNA kit (Illumina) was performed, followed by 2 × 150 bp paired-end sequencing on a NovaSeq 6000 platform.

Paired reads from each sample were analyzed using ViPER, a bioinformatic pipeline designed to trim and assemble paired-end Illumina reads and classify the resulting contigs (6).

Thereafter, trimmed reads were mapped to the B19 reference genome (B19-J35, AY386330) using bwa-mem2. Mapping results were obtained with samtools coverage (7), and fasta consensus sequences were generated using samtools mpileup and ivar consensus (8, 9). All tools were run with default parameters unless otherwise specified.

The reference mapping yielded full genome coverage (5596 nt) for four samples (LP1, LP2, LP4, and LP5) (see Table 1). All the sequences shared a nucleotide identity higher than 98% with the reference genome, suggesting that they belong to genotype 1. The GC content ranged between 43.6% and 43.9%.

**TABLE 1:** Mapping results and analysis of coding regions compared to reference strain B19-J35 (AY386330)

Specimens			LP1	LP2	LP4	LP5
Nucleoditic sequences (mapping results)	Number of trimmed reads aligned to the reference		3,454,461	400,427	47,893,753	3,968,351
	Covered bases (%)^*a*^		100	100	100	100
	Mean depth of coverage		77,100	7,530	1,060,210	87,800
	Mean baseQ in covered region		36.1	36.3	36	35.9
Proteins	Nonstructural protein (NS1) (672 nucleotides)	Coverage (%)	100	100	100	100
		Concordance (%)	99.3	99	99.3	99.8
		AA^*c*^ changes	L8I, L57F, A71V, F111L, S190G, T505P, F554S	L8I, L57F, A71V, F111L, S190G, S195P, D280N, T505P, F554S	L57F, A71V, F111L, S190G, S344T, T505P, F554S	C17S, L57F, A71V, F111L, E114G, G159A, T163N, I181M
	7.5 kDa protein (75 nucleotides)	Coverage (%)	100	100	100	100
		Concordance (%)	100	98.7	100	100
		AA changes	None	M1T^*b*^	None	None
	Capsid protein 1 (782 nucleotides)	Coverage (%)	100	100	100	100
		Concordance (%)	99.9	99.9	99.9	99.3
		AA changes	E14K	E14K	E14K	D12N, E14K, V30L, S98N, D107N, A260T, N533S
	Protein X (82 nucleotides)	Coverage (%)	100	100	100	100
		Concordance (%)	100	100	100	99.4
		AA changes	None	None	None	A15T
	Capsid protein 2 (555 nucleotides)	Coverage (%)	100	100	100	100
		Concordance (%)	100	100	100	99.7
		AA changes	None	None	None	A33T, N306S
	11 kDa protein (95 nucleotides)	Coverage (%)	100	100	100	100
		Concordance (%)	100	99.2	100	98.9
		AA changes	None	T61K	None	M1T^*b*^, I54V

^*a*^ Few nucleotidic positions in the terminal repeats (59, 15, and 19 in LP1, LP4, and LP5, respectively) were covered by less than 10 reads and were, therefore, removed (external nucleotides) or labeled as “N” (internal nucleotides) in the consensus sequences. A G insertion was observed at nt 567 in the LP5 5’ TR.

^*b*^ A mutation in the start codon (resulting in the M1T amino acid change according to the reference) was observed for the 7.5 kDa protein in LP2 and the 11 kDa protein in LP5, and new coding DNA sequences (CDS) were defined using the NCBI ORFfinder.

^*c*^ AA: amino acid.

The coding DNA sequences shared 98.7–100% identity with the reference. CDS features were defined by pairwise alignment with the reference. The highest number of amino acid substitutions was observed in nonstructural protein 1 (NS1) for all isolates (7, 9, 7, and 8 for LP1, LP2, LP4, and P5, respectively). Regarding the structural proteins, only the capsid protein 1 substitution E14K was observed in the LP1, LP2, and LP4 genomes, while LP5 displayed 7 and 3 mutations in the capsid proteins 1 and 2, respectively.

## Data Availability

All raw reads have been submitted to NCBI’s SRA at PRJNA979336. BioSample accessions are: LP1: SAMN35578145, LP2: SAMN35578146, LP4: SAMN35578147, LP5: SAMN35578148. The consensus sequences have been deposited in GenBank under the accession nos. OR138119, OR138120, OR138121, OR138122.
